# Metabolite Variations in *Lepidium sativum* Seeds Among Different Germplasms: Unraveling the Link Between Environment and Chemical Compounds

**DOI:** 10.1002/fsn3.72177

**Published:** 2026-08-06

**Authors:** Seyed Mehdi Talebi, Majid Mahdieh, Ghassan Mohammad, Mansour Ghorbanpour

**Affiliations:** ^1^ Department of Biology, Faculty of Science Arak University Arak Iran; ^2^ Department of Medicinal Plants, Faculty of Agriculture and Natural Resources Arak University Arak Iran

**Keywords:** chemotype variation, environmental influence, essential oil composition, garden cress, gas chromatography, phytochemistry

## Abstract

This research investigated the intraspecific phytochemical diversity in garden cress by analyzing the seed essential oils, fatty acid composition, and antioxidant activity of seed oils in various cultivated populations. The goal was to understand how phytochemical variations occur across populations and how these variations affect their biological activities. To conduct this study, approximately 500 g of seeds were harvested from each population across ten different locations. Essential oils were extracted using the hydrodistillation method with a Cleavinger‐type apparatus, while seed oils were obtained through solvent extraction using a Soxhlet apparatus. Antioxidant activity was determined using the DPPH method. The resulting essential oils and seed oils were then analyzed with an Agilent gas chromatograph (GC) and gas chromatography–mass spectrometry (GC/MS) to identify their chemical profiles. Appropriate internal standards were used to enhance the accuracy of the analyses. The examined populations were distributed across the Holarctic and Paleotropical kingdoms, encompassing several floristic districts, indicating wide ecological adaptability. Essential oil yield was low and showed no significant difference among populations; however, it was significantly influenced by environmental factors, with positive correlations to habitat humidity and northern distribution and a negative correlation with solar radiation. Chemical analysis revealed hydrocarbon dominance in all populations, with benzyl nitrile identified as the major constituent, although its concentration varied considerably. Secondary constituents such as benzyl isothiocyanate, benzaldehyde, and other minor compounds showed population‐specific variation. Multivariate analyses indicated distinct chemotypic groupings, although these were only weakly associated with geographic or phytogeographic factors. Seed oil fatty acid profiles were dominated by unsaturated fatty acids, particularly α‐linolenic acid, with minor variation among populations, indicating relative metabolic stability. Antioxidant activity assessed by the DPPH assay was weak across all populations, with no significant pairwise differences detected. Overall, the results suggest that while essential oil yield is environmentally sensitive, core chemical composition and fatty acid profiles remain relatively stable. These findings highlight the combined influence of ecological conditions and intrinsic regulation on phytochemical variability in *L. sativum.*

## Graphical abstract

Comparative phytochemical analysis of *Lepidium sativum* seed populations revealed environmentally driven variation in essential oil composition, while fatty acid profiles remained largely stable across populations. Benzyl nitrile was the dominant essential oil constituent, α‐linolenic acid was the major fatty acid, and all populations exhibited weak antioxidant activity, highlighting the combined influence of environmental conditions and intrinsic metabolic regulation on phytochemical diversity.

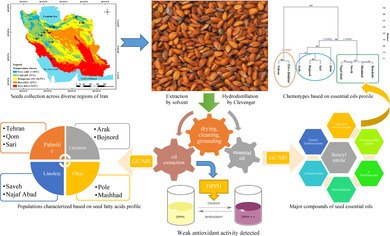

## 1 Introduction

Abiotic environmental factors strongly influence the biosynthesis and accumulation of plant secondary metabolites, which are essential for plant defense and adaptation. Variations in geographic location, climate, and phytogeographic distribution can significantly alter the composition and concentration of compounds such as essential oils and fatty acids (Pant et al. 2021; Qaderi et al. 2023). Similarly, agroecological conditions—including soil type, irrigation, light, temperature, plant density, cultivation practices, and nutrient availability—can lead to intraspecific differences in phytochemical profiles, affecting both plant survival and the medicinal, nutritional, and industrial value of plant‐derived products (Semenko and Pospelov 2023; Zhao et al. 2025; Tian et al. 2025). In cruciferous plants (Brassicaceae family), secondary metabolites also vary widely among species, populations, developmental stages, and plant organs, and are strongly shaped by environmental conditions (Gmižić and Šola 2025). For example, factors such as temperature, water stress, metal exposure, soil characteristics, and cultivation systems can induce significant phytochemical variation in species of *Brassica* L. and *Sinapis* L. (Aghajanzadeh et al. 2019; Biondi et al. 2021; Ayadi et al. 2022).

The Brassicaceae family, also known as the cruciferous plants or mustard family, is a major contributor to human food sources and is considered one of the most economically important plant families. These vegetables are known for their nutritional value and the presence of beneficial secondary metabolites such as vitamins (e.g., C, E, and folic acid), carotenoids, flavonoids, phenolic compounds, essential oil, fatty acids, and glucosinolates (Toro et al. 2024).

This family is characterized by its herbaceous nature, though some members are shrubs. Key features include simple leaves in alternate or rosette arrangement, lack of stipules, terminal inflorescences without bracts, flowers with four free sepals and petals, and two short and four long free stamens. The fruits are typically siliques or silicles (APG III 2009). Plant‐based materials, especially those from the Brassicaceae family, are considered important in cancer prevention and may also contribute to cancer treatment. Regular consumption of these vegetables is associated with a reduced risk of certain types of cancer, largely due to the presence of bioactive compounds called isothiocyanates (ITCs). These ITCs are formed when plant tissues are damaged, triggering the enzymatic hydrolysis of glucosinolates by the enzyme myrosinase. This reaction produces compounds with potential anticancer properties, highlighting the nutritional and therapeutic significance of Brassicaceae vegetables (Dinh et al. 2021).

*Lepidium sativum* L. is a fast‐growing annual herb from the Brassicaceae family. It typically grows to a height of 15–45 cm, bearing small white or pinkish flowers and producing obovate to broadly elliptic siliques containing reddish‐brown seeds. Native to Europe and southwestern Asia, it's now cultivated worldwide. The plant is known for its pungent, peppery flavor and is commonly called garden cress, pepper grass, or pepperwort (Shah et al. 2021).

Various parts of *L. sativum* , including the leaves, flowers, and seeds, contain diverse secondary metabolites with valuable biological activities, such as antihypertensive, anti‐inflammatory, antiasthmatic, immunomodulatory, and antioxidant properties (Vazifeh et al. 2022; Aboul‐Naser et al. 2024). Owing to these pharmacological attributes, *L. sativum* has traditionally been used in herbal medicine for the treatment of various disorders, including asthma, liver ailments, bronchitis, cough, bleeding piles, and scurvy (Shah et al. 2021; Yadav et al. 2022).

Experimental investigations have shown that the essential oil of *Lepidium meyenii* Walp., which contains a high concentration of benzyl nitrile, exhibits strong insecticidal activity against the larvae, nymphs, and adults of *Haemaphysalis longicornis* Neumann. Benzyl nitrile, in particular, has been identified as a key bioactive compound responsible for this enhanced effectiveness, demonstrating greater insecticidal activity against larval and nymphal stages compared to other conventional insecticides (Lee and Lee 2025).

A previous study (Musara et al. 2020) reported that the seed oil of *L. sativum* exhibits favorable physical properties, including its color, odor, specific gravity, refractive index, and viscosity, making it suitable for use in food product development and beverage fortification. The oil contains a balanced composition of saturated and unsaturated fatty acids, including palmitic acid (as a saturated fatty acid) as well as oleic acid, linoleic acid, and tocopherols (unsaturated and bioactive compounds), highlighting its nutritional and functional value (Shah et al. 2021; Khophloiklang et al. 2024; Aragie et al. 2025).

Previous studies on garden cress have revealed substantial variations in its infraspecific morphological traits, geographic distribution (Bedassa et al. 2013; Sabbaghnia et al. 2024), and genetic structure (Bansal et al. 2012). However, there has been a lack of comprehensive phytochemical diversity investigation within this species. To address this gap, the study was conducted on 10 cultivated populations of *L. sativum* in Iran, focusing on seed essential oil composition, fatty acid profiles, and antioxidant activity of the seed oil.

Iran, with an area exceeding 1,648,000 km^2^ is located in Southwest Asia, is characterized by its diverse climate zones, phytogeographic regions and serves as a vital bridge for plant migration between eastern and western Eurasia. The Iranian plateau, a large portion of the country, is recognized as a hotspot of evolutionary and biological diversity (Noroozi et al. 2019). Within its borders, six distinct climate zones are found: mild and humid (Caspian Sea region), cool and sub‐humid (western Iran), cold and temperate semi‐arid (northwestern Iran), warm and semi‐arid (southwestern Iran), cool and arid (northeastern Iran), and warm and hyperarid (southeastern and central Iran) (Najafi and Alizadeh 2023). Therefore, it is crucial to investigate the phytochemical variations across different populations of the same species growing throughout Iran.

The specific objectives of this study were to (1) characterize the essential oil composition of *L. sativum* seeds from 10 cultivated populations, (2) determine the fatty acid profile of seed oils, (3) assess their antioxidant activities, and (4) identify potential chemotypic variations among populations. To the best of our knowledge, no previous study has comprehensively investigated the phytochemical variability and antioxidant activity of *L. sativum* seed oil across different populations.

## 2 Material and Methods

### 2.1 Plant Materials

**FIGURE 1:**
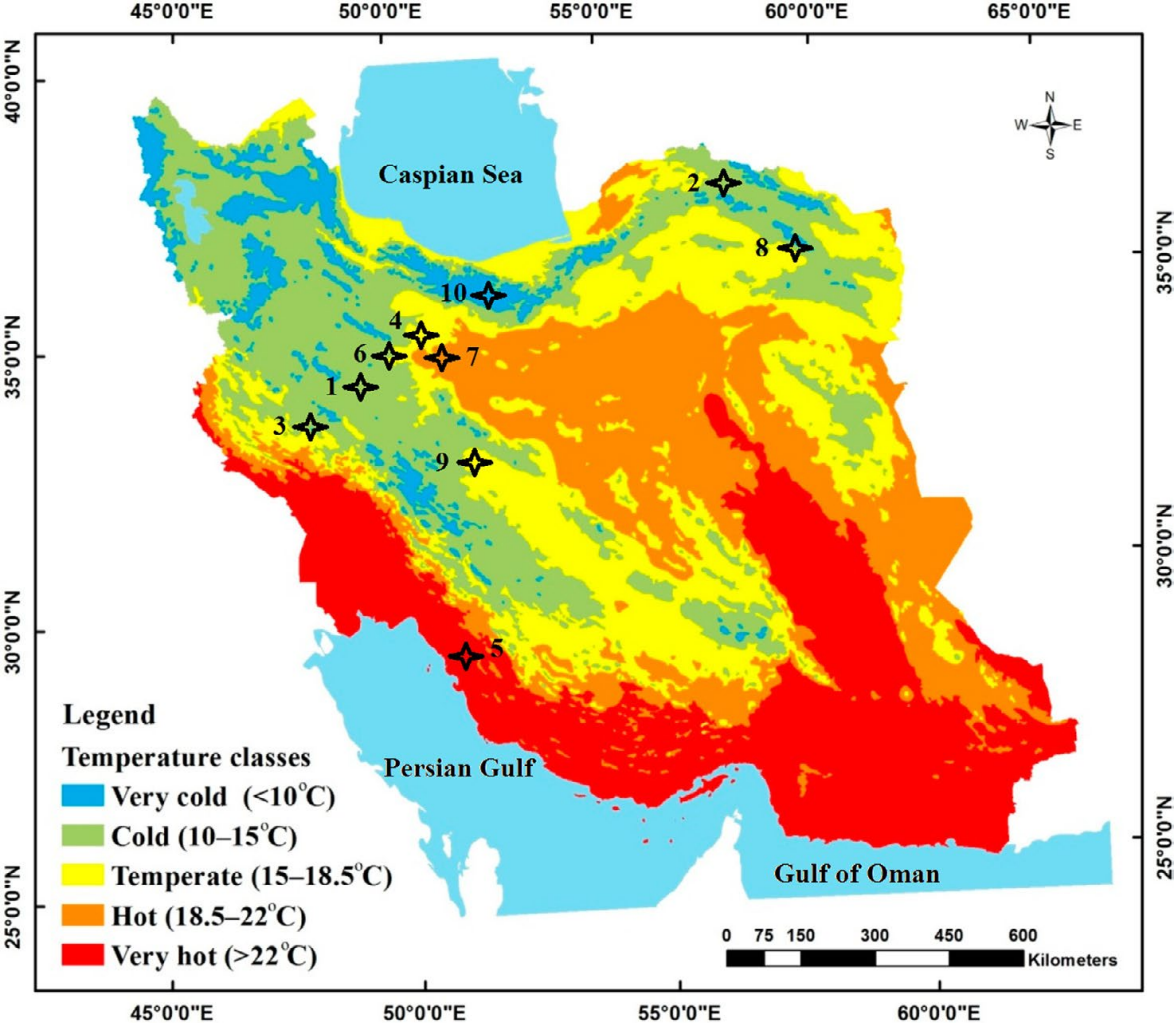
Distribution map of the examined *L. sativum* populations. Asterisks and numbers indicate the locality of the populations, as follows: listed in Table 1. The base map was adapted from Asadi‐Oskouei et al. (2022) with minor modifications.

Seed samples from 10 cultivated populations of *Lepidium sativum* were collected from different regions of Iran at the end of summer 2024. Voucher specimens were deposited in the Herbarium of Arak University (Table 1; Figure 1). From each population, approximately 500 g of intact, mature seeds were selected for the assessment of antioxidant activity, analysis of seed essential oil composition, and determination of seed oil fatty acid profiles.

**TABLE 1:** The locality addresses of the evaluated *L. sativum* populations in Iran.

Populations	Localities addresses	Geographical coordinates			Climatic features						Phytogeography	Voucher no.
		Latitude (N)	Longitude (E)	Altitude (m)	Max Tem. (°C)	Min. Tem (°C)	Average tem (°C)	Precipitation (mm)	Humidity (%)	Solar radiation (kWh/m^2^)		
Arak	Markazi province	34°08′73″	49°70′22″	1718	18.2	4.67	11.41	147.43	53.66	5.6	Kurdo‐Zagrosian	AUH 6980
Bojnurd	North Khorasan province	37°47′45″	57°32′33″	1070	21.5	9.4	16.01	95.11	55	5.4	Khorassanian	AUH 6981
Pol‐e‐Dokhtar	Lorestan province	33°14′50″	47°72′23″	659	21.34	6.47	13.88	166	46	5.6	Kurdo‐Zagrosian	AUH 6982
Tehran	Tehran Province	35°72′19″	51°33′47″	1200	19.84	8.08	13.93	113.04	62	5.5	Atropatenian	AUH 6983
Bousher	Bousher province	28°91′45″	50°82′79″	18	29.06	18.15	23.85	40.59	32.66	6.1	Nubo‐sindian	AUH 6984
Saveh	Markazi province	35°02′40″	50°35′49″	1000	20.3	8.57	14.46	125.11	59.32	5.9	Central Iranian	AUH 6985
Qom	Qom province	34°64′16″	50°87′46″	936	24.03	11.36	17.67	94.47	62.66	5.6	Central Iranian	AUH 6986
Mashhad	Khorasan Razavi province	36°29′72″	59°60′67″	995	23.38	10.6	17.47	90.13	45	5.7	Khorassanian	AUH 6987
Najaf Abad	Isfahan province	32°63′72″	51°35′73″	1200	24.44	10.55	17.47	67.11	46.66	5.9	Central Iranian	AUH 6988
Sari	Mazandaran province	36°56′59″	53°05′86″	35	18.58	8.21	13.37	111.96	72	4.0	Exino‐Hyrcanian	AUH 6989

*Note:* Max Tem. Seasonal average maximum temperature, Min. Tem Seasonal average minimum temperature, Seasonal average mean temperature, and seasonal precipitation.

### 2.2 Ecology and Phytogeography

The climatic characteristics of the populations' habitats, including seasonal precipitation (mm), average maximum, minimum, and mean temperatures (°C), humidity (%), and solar radiation (kWh/m^2^) for the period 1990–2022, were obtained from the Islamic Republic of Iran Meteorological Organization (www.irimo.ir). The geographic coordinates of the habitats (altitude, longitude, and latitude) were determined using a Garmin eTrex 10 GPS. Zohar's phytogeographic mapping system (Zohary 1973) was utilized to classify the phytogeographical distribution of plant populations based on their habitats.

### 2.3 Essential Oil Extraction

Essential oils were isolated from seed samples by hydrodistillation using a Clevenger‐type apparatus in accordance with the European Pharmacopeia (2008). The extracted oils were dried over anhydrous sodium sulfate (CAS No. 7757‐82‐6, Sigma‐Aldrich) to remove residual moisture and subsequently stored at 4°C until analysis. All extractions were performed in triplicate for each population.

Prior to extraction, 500 g of seeds were air‐dried at room temperature (20°C–25°C) for 36 h under ambient laboratory conditions to reduce moisture content while preserving volatile constituents (Prakash et al. 2026). During this process, samples were spread in a thin, uniform layer to ensure adequate air circulation and to minimize microbial contamination. The dried seeds were then ground using a laboratory mill to obtain a fine powder, thereby increasing surface area and enhancing extraction efficiency (Hundie et al. 2023).

Ground seed material from each population was subjected to hydrodistillation for 3 h. The collected essential oils were subsequently dried again over anhydrous sodium sulfate to ensure complete removal of water. The purified essential oils were transferred into labeled glass vials, tightly sealed, and stored under refrigeration at 4°C until further analysis (Sareriya et al. 2023; Sander et al. 2025).

Essential oil yield of each population was calculated as a percentage according to the following equation:

$$\text{Yield}=\frac{V}{m}\times 100$$

where *V* represents the volume of the extracted essential oil (mL), and m denotes the mass of the dry plant material used for extraction (g) (Sander et al. 2025).

### 2.4 Oil Extraction and Fatty Acid Methyl Ester Preparation

About 2 g of mature garden cress seeds were processed to extract oil. The seeds were dried, cleaned, ground, and cooked. An automated extraction system (FOSS Soxtec 2055) was used, with n‐hexane (Sigma‐Aldrich) as the solvent. After initial boiling at 70°C for 25 min (immersion phase), the samples underwent rinsing cycles for 30 min, followed by solvent recovery for 15 min and final drying at 90°C for 10 min. The extraction procedure was repeated three times to improve oil recovery.

The extracted *L. sativum* seed oils were transferred to a three‐neck round‐bottom flask and preheated at 70°C for 30 min. A methanolic sodium hydroxide solution (Sigma‐Aldrich) was subsequently added, and the reaction mixture was agitated at 500 rpm at a constant temperature of 65°C. Following the transesterification of the *L. sativum* seeds oil, the mixture was transferred to a separating funnel and allowed to settle for 24 h. This gravity‐driven separation isolates the dense glycerol byproduct from the upper fatty acid methyl ester (FAME) phase. The FAME fraction was collected and washed with warm distilled water (approximately 50°C) to eliminate residual impurities, including unreacted methanol, catalyst, soaps, and trace glycerol. Finally, residual water and methanol were removed using a rotary evaporator at 70°C, yielding the purified ester phase (Zamba and Reshad 2022; Shariatzadeh et al. 2025).

### 2.5 GC and GC–MS Analyses

Gas chromatography (GC, Agilent‐USA) and gas chromatography–mass spectrometry (GC–MS, Agilent Technologies‐ USA) were employed to determine the fatty acid profiles of seed oils and the chemical composition of seed essential oils in different *L. sativum* populations.

For GC analysis, separation was performed on an HP‐5 capillary column (30 m × 0.32 mm × 0.25 μm) using helium as the carrier gas at a constant flow rate of 1.5 mL/min. The oven temperature was initially set at 150°C, increased to 280°C at a rate of 3°C/min, and held at 280°C for 5 min. Samples (1 μL) were injected at 250°C with a split ratio of 50:1. Seed oil samples were diluted in hexane (1:20, v/v), whereas essential oil samples were diluted in methanol (1:20, v/v). GC–MS analysis was conducted using an HP‐5MS capillary column (30 m × 0.25 mm × 0.25 μm) with helium as the carrier gas at a constant flow rate of 1.0 mL/min. The oven temperature program was set from 150°C to 260°C at a rate of 3°C/min, followed by a 5 min hold at 260°C. A 1 μL aliquot of each diluted sample was injected at 250°C with a split ratio of 30:1. Electron ionization (EI) was carried out at 70 eV, and mass spectra were acquired in scan mode over a mass range of 40–400 m/z (Shariatzadeh et al. 2025).

The chemical constituents were identified by comparing their mass spectra with those stored in the library (Adams 1995) and library of Wiley 7 mass spectral (NIST 17‐Wiley and Sons 2017). To ensure accuracy, the experimental Retention Indices (RI) and Kovats Indices (KI) were calculated for all compounds using a homologous series of n‐alkanes (C_3_–C_40_) injected under the same analytical conditions. These calculated RI and KI were then compared with values reported in the mentioned literature for an HP‐5 column (Wesołowska et al. 2016; Sander et al. 2025). Compounds were reported only when a minimum match factor (similarity index > 80%) was achieved, and experimental RIs deviated by no more than ±10–15 units from the literature values. Certain major components were further confirmed via the injection of authentic analytical standards under identical conditions. The relative percentages of detected compounds were calculated by normalization of the gas chromatography area (Kumar et al. 2017; Maurya et al. 2021, 2022). The fatty acid methyl ester (FAME) types in extracted oils were determined by comparing their retention index (RI) in gas chromatography (GC) with known FAME standards, typically purchased from Sigma‐Aldrich (Ltd). This method allows for accurate identification of different FAMEs by comparing their elution times in a GC column. Specifically, standards of Arachidic acid; 39,383, Behenic acid; 11,909, Eicosadienoic; E3127, Erucic acid; 45,629, Heptadecenoic acid; H8896, Lignoceric acid; L6641, Linoleic acid; 62,230, γ + Linolenic acid; 62,174, Margaric acid; H3500, Myristic acid; 70,079, Myristoleic acid; 41,788, Oleic acid; 75,090, Palmitic acid; 76,119, Palmitoleic acid; 76,169, and Stearic acid; 85,679 were used.

### 2.6 DPPH Radical Scavenging Assay

To determine the free radical scavenging activity, a DPPH (2,2‐diphenyl‐1‐picrylhydrazyl‐CAS no. 1898‐66‐4, Sigma Aldrich) radical scavenging method is used according to Blois (1958) and Desmarchelier et al. (1997). 0.1 mM solution of DPPH in methanol was prepared, and 2.4 mL of this solution was mixed with 1.6 mL of extracted oil of each population in methanol at diverse concentrations (12.5–150 μg/mL). After 35 min. incubation in a dark at room temperature, the absorbance of the mixture is measured at 517 nm. BHT was used as reference. The percentage DPPH radical scavenging activity is then calculated using a specific following formula (comparing the absorbance of the sample to a control): DPPH radical scavenging activity % = $\frac{A0-A1}{A0}$ × 100, where A_0_ is the absorbance of the control, and A_1_ is the absorbance of the oil samples. The outputs are then plotted against various concentrations of the extracted oils, and the IC50 (Inhibitory Concentration 50) is calculated from the graph. This process is repeated three times for each concentration to ensure accuracy and reliability of the data.

### 2.7 Statistical Analyses

Prior to analysis, data of seeds essential oil composition and fatty acid profile of all populations were expressed as relative percentages of total composition and treated as compositional data. To address the constant‐sum constraint and ensure suitability for multivariate analyses (such as PCA, Pearson correlation, and clustering) centered log‐ratio (clr) transformation was applied. For ANOVA, assumptions of normality and homogeneity of variances were evaluated, and when necessary, appropriate transformations (e.g., arcsine square root or log transformation) were performed (Kaur et al. 2026).

The UPGMA (Unweighted Pair Group Method with Arithmetic Mean) method based on Euclidean distance was used to measure the dissimilarity between populations. It is a hierarchical clustering algorithm used to construct a dendrogram, which visually represents the relationships between populations (Yoshida et al. 2022). Moreover. Principal Component Analysis (PCA) is used to group populations based on their variable phytochemical characteristics (all quantitative data). These analyses were performed using PAST (PAleontological Statistics) ver. 4.03 software.

Principal Component Analysis (PCA) is a dimensionality reduction technique that transforms a dataset with a large number of correlated variables into a smaller set of uncorrelated variables known as principal components. These components are ranked according to the proportion of variance they explain, thereby enabling the identification of the most significant patterns within the data. In this context, the first two principal components, which account for the highest variance and possess the largest eigenvalues, are typically selected for constructing the PCA plot. This graphical representation facilitates the visualization of relationships among samples and allows for the identification of clusters of populations exhibiting similar phytochemical profiles (Jolliffe and Cadima 2016).

SPSS ver. 15 was used to perform the Pearson bivariate correlation coefficient to analyze the relationships between seed fatty acid profiles, essential oil yield and composition with geographic/climatic data of populations. Analysis of variance (ANOVA) was used to evaluate differences among variables at a significance level of *p* ≤ 0.05. For traits showing significant variations, *post hoc* multiple comparison tests, including Duncan's multiple range test, Tukey B, and Tukey's honestly significant difference (HSD) test, were applied. While ANOVA identifies the presence of overall differences among group means, it does not specify which groups differ. Therefore, post hoc analyses were conducted to perform pairwise comparisons between groups while controlling for Type I error, thereby enabling precise identification of the sources of variation among populations (Kim 2015).

## 3 Results

### 3.1 Phytogeographic Study

The examined populations were distributed across two distinct phytogeographic kingdoms: the Holarctic and the Paleotropical. The Bushehr population belonged to the Nubo‐Sindian district of the Paleotropical kingdom. In contrast, the Holarctic populations were associated with the Irano‐Turanian and Euro‐Siberian regions. Specifically, the Sari population was assigned to the Exino‐Hyrcanian district of the Euro‐Siberian region. The remaining populations were classified within different districts of the Irano‐Turanian region, including: the Kurdo‐Zagrosian district (Arak and Pol‐e‐Dokhtar populations), the Kopet Dagh district (Bojnurd population), the Atropatenian district (Tehran population), and the Central Iranian district (the remaining populations).

### 3.2 Seed Essential Oil Yield and Composition

The essential oil yield, composition, Kovats indices (KI), and retention times (RT) of compounds of the evaluated populations are presented in Table 2.

**TABLE 2:** Essential oils composition of the evaluated *L. sativum* populations.

Components/populations	RI	KI	Arak	Bojnord	Pol‐e‐Dukhtar	Tehran	Bousher	Saveh	Qom	Mashhad	Najaf Abad	Sari
Essential oil yield (% v/w) ^ns^			0.17 ± 0.015	1.9 ± 0.02	0.19 ± 0.025	0.25 ± 0.018	0.15 ± 0.020	0.16 ± 0.032	0.19 ± 0.025	0.22 ± 0.026	0.14 ± 0.026	0.27 ± 0.020
4‐Methyl‐3‐penten‐2‐one	5.83	801	0.22	—	—	0.60	—	1.65	2.71	—	—	—
3‐Methylbutanenitrile	7.66	849	—	0.82	—	—	—	—	—	—	—	0.93
Ethylbenzene	8.24	856	—	0.64	—	—	—	—	—	—	—	0.72
Styrene	9.27	915	—	0.45	—	—	—	—	—	—	—	0.60
Bicyclo(3.2.1)octan‐3‐one	12.29	950	—	0.19	—	—	—	—	—	—	—	0.25
Propylbenzene	12.62	962	—	0.71	—	—	—	—	—	—	—	0.83
2‐Propylthiophene	12.78	966	—	—	—	—	—	—	—	—	—	0.37
Benzaldehyde	13.50	975	6.22	5.37	1.80	1.10	2.27	0.50	2.51	1.00	0.81	6.01
Limonene	16.46	1025	—	—	—	0.13	—	—	—	—	—	—
1‐ethenyl‐2‐methylbenzene	16.60	1035	—	—	—	—	—	—	—	—	—	0.16
1‐Pentyl‐1H‐pyrrole	17.78	1058	—	—	—	—	—	—	—	—	—	0.42
n‐Butylbenzene	17.92	1068	—	2.43	—	—	—	—	—	—	—	2.48
n‐Octanol	18.81	1069	—	—	—	0.28	—	—	0.05	—	—	—
Benzene, 2‐butenyl	19.13	1084	—	0.31	—		—	—		—	—	0.31
Benzenemethanethiol	19.66	1094	3.01	2.23	0.50	1.50	0.58	0.51	4.54	1.59	0.38	2.48
n‐Undecane	19.87	1100	—	0.23	—	—	—	—	—	—	—	0.28
Benzoic acid, methyl ester	20.25	1109	—	0.36	—	—	—	—	—	—	—	0.34
Benzene, 2‐butenyl—	21.59	1133	—	—	—	—	—	—	—	—	—	2.64
Benzyl nitrile	22.98	1143	81.35	77.44	51.25	52.05	95.68	96.33	68.20	95.43	97.31	74.88
Pentylbenzene	23.07	1159	—	1.63	—	—	—	—	—	—	—	1.49
trans‐1‐Phenyl‐1‐pentene	23.71	1175	—	0.15	—	—	—	—	—	—	—	0.15
Benzyl methyl sulfide	23.83	1178	—	0.23	—	—	—	—	—	—	—	0.22
Benzeneacetic acid, methyl ester	24.32	1188	—	—	—	—	—	—	—	—	—	0.19
1‐(2‐Furanylmethyl)‐1H‐pyrrole	24.49	1199	0.15	—	—	—	—	—	—	—	—	0.17
Hexylbenzene	28.00	1254	—	0.43	—	—	—	—	—	—	—	0.43
Carvacrol	30.12	1299	—	—	0.52	—	—	—	—	—	—	—
Thiocyanic acid, phenylmethyl ester	32.47	1335	—	—	0.85	0.91	—	—	—	—	—	—
Heptylbenzene	32.61	1336	—	0.36	—	—	—	—	—	—	—	0.37
Benzyl Isothiocyanate	33.28	1377	7.75	—	41.70	42.67	0.55	0.41	1.57	0.20	0.19	0.25
Benzyl methyl disulfide	34.16	1402	0.15	0.69	0.30	—	0.20	—	—	—	—	0.56
Isopropyl tetradecanoate	50.41	1829	—	—	—	—	—	—	6.05	—	—	0.14
n‐Nonadecane	52.85	1900	—	—	—	—	—	—	5.44	—	—	0.14
n‐Eicosane	56.08	2000	—	—	—	—	—	—	0.68	—	—	—
Benzyl disulfide	59.01	2070	0.30	1.07	2.71		0.52	0.30	0.70	1.58	1.21	1.03
n‐Heneicosane	59.19	2100	—	—	—	0.35	—	—	7.03	—	—	—
Monoterpene hydrocarbons			—	—	—	0.13	—	—	—	—	—	—
Oxygenated monoterpenes			—	—	0.52	—	—	—	—	—	—	—
Sesquiterpene hydrocarbons			—	—	—	—	—	—	—	—	—	—
Oxygenated sesquiterpenes			—	—	—	—	—	—	—	—	—	—
Other compounds			99.15	95.74	99.11	99.46	99.80	99.70	99.48	99.80	99.90	98.84
Total			99.15	95.74	99.63	99.59	99.80	99.70	99.48	99.80	99.90	98.84

*Note:* All the values of components are in %. Abbreviations: KI, Kovats index; ^ns^, No significant variation; RI, Retention index; V/W %, volume–weight ratio in percentage.

The overall essential oil yield obtained from the seeds of the examined populations was very low. The highest yield was recorded for the Sari population (0.27% ± 0.020% v/w), whereas the lowest yield (0.14% ± 0.026% v/w) was observed in the Njaf Abad population. Analysis of variance (ANOVA) revealed no significant difference (*p* ≤ 0.05) in essential oil yield among the populations (Table 3).

**TABLE 3:** Results of ANOVA test on essential oil yield and antioxidant activity of seed oils, and post hoc tests on antioxidant activity.

ANOVA test	Sum of squares	df	Mean square	*F*	Sig.
Essential oil yield					
Between Groups	0.002	9	0.000	0.510	0.850
Within Groups	0.010	20	0.000		
Total	0.012	29			
Antioxidant activity					
Between Groups	254625.227	9	28291.692	16.984	0.0000
Within Groups	33315.661	20	1665.783		
Total	287940.887	29			

Post hoc tests		1	2	3	4	5
Tukey HSD^a^						
6	3	7.497500E				
9	3	8.047400E	8.0474002			
4	3	8.3193002	8.319300E	8.319300E2		
10	3	8.4260002	8.4260002	8.426000E2		
8	3	8.4332002	8.4332002	8.433200E2		
2	3	8.5748002	8.5748002	8.574800E2		
5	3		8.7697002	8.769700E2		
7	3		9.1849002	9.184900E2		
3	3			9.329100E2		
1	3				1.10913	
Sig.		0.092	0.065	0.134	1.000	
Tukey B^a^						
6	3	7.4975002				
9	3	8.0474002	8.0474002			
4	3	8.3193002	8.3193002	8.319300E2		
10	3	8.4260002	8.4260002	8.426000E2		
8	3	8.4332002	8.4332002	8.433200E2		
2	3	8.5748002	8.574802	8.574800E2		
5	3		8.7697002	8.769700E2		
7	3			9.184900E2		
3	3			9.329100E2		
1	3				1.10913	
Duncan^a^						
6	3	7.4975002				
9	3	8.047402	8.0474002			
4	3		8.3193002			
10	3		8.4260002	8.426000E2		
8	3		8.43320E2	8.433200E2		
2	3		8.57480E2	8.574800E2		
5	3		8.7697002	8.769700E2	8.76970	
7	3			9.184900E2	9.18490	
3	3				9.32910	
1	3					10E3
Sig.		0.115	0.068	0.052	0.127	1.000

*Note:* Means for groups in homogeneous subsets are displayed.

^a^ Uses Harmonic Mean Sample Size = 3.000.

Hydrocarbons constituted the predominant class of essential oil components in all populations. Only trace amounts of monoterpene hydrocarbons–represented by limonene (0.13%) in the Tehran population– and oxygenated monoterpenes–represented by carvacrol (0.52%) in the Pol‐e‐Dokhtar population–were detected.

Benzyl nitrile was identified as the major constituent in all populations, ranging from 51.25% in the Pol‐e‐Dokhtar population to 97.31% in the Najaf Abad population. The second major constituent varied among populations and included: benzyl isothiocyanate (0.19%–41.70%) in the Arak, Pol‐e‐Dokhtar, and Tehran populations; benzaldehyde (0.50%–6.22%) in the Bojnurd, Bushehr, and Sari populations; benzyl disulfide (0.30%–2.71%) in the Najaf Abad population; benzenemethanethiol (0.38%–4.54%) in the Mashhad population; isopropyl tetradecanoate (0.14%–6.05%) in the Qom population; and 4‐methyl‐3‐penten‐2‐one in the Saveh population. In addition, the examined populations exhibited distinct profiles of trace compounds in their essential oils. No significant correlation was observed between the major essential oil constituents and the geographic locations or climatic conditions of the populations.

**FIGURE 2:**
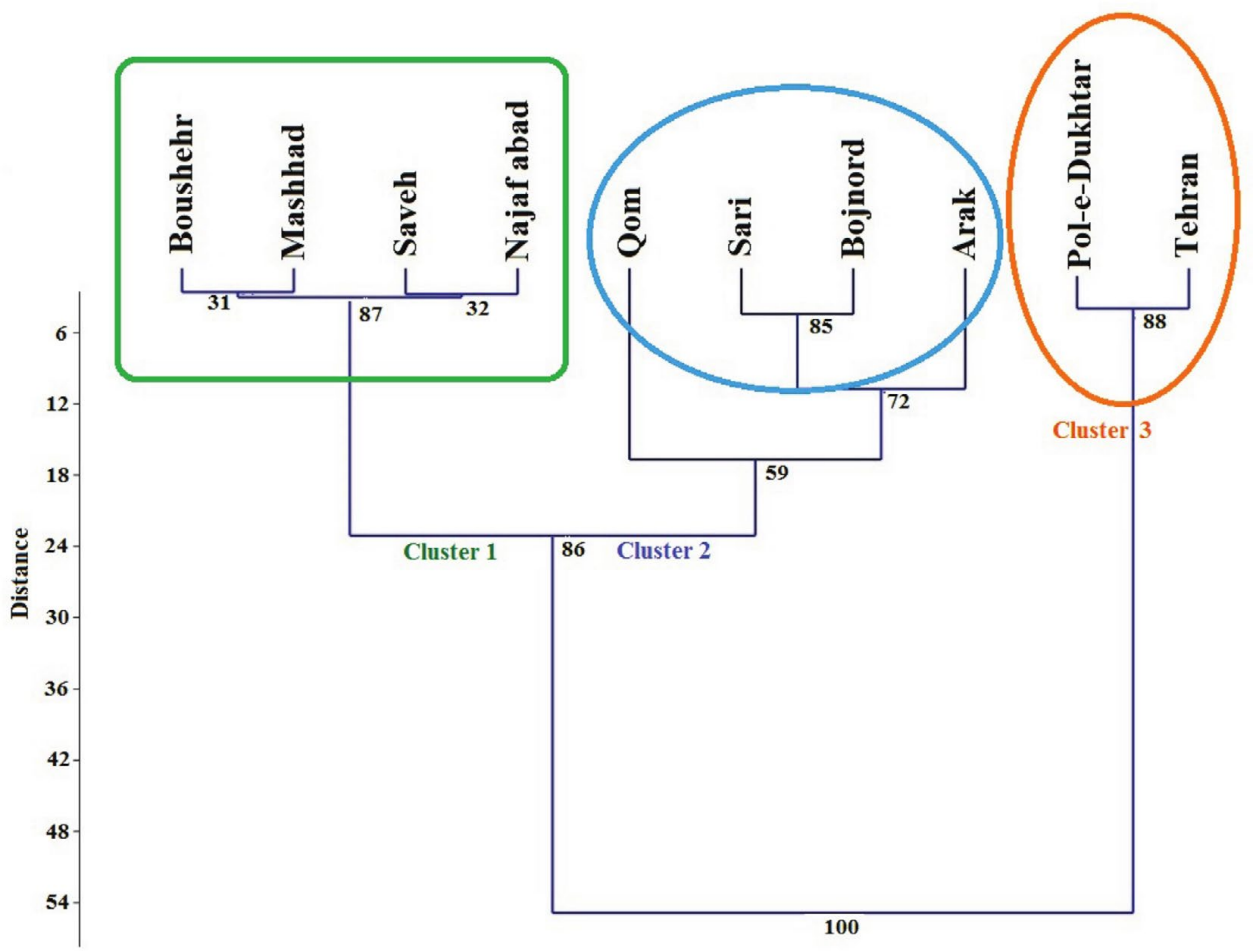
The UPGMA clustering of the examined *L. sativum* populations based on the seed essential oils composition (number below each node exhibit a bootstrapping value).

Based on UPGMA clustering analysis (Figure 2), the examined populations were grouped into three major clusters: (1) Bushehr, Mashhad, Saveh, and Najaf Abad populations, with a bootstrap support value of 87%; (2) Qom, Sari, Bojnurd, and Arak populations; and (3) Tehran and Pol‐e‐Dokhtar populations, with a bootstrap support value of 100%.

**FIGURE 3:**
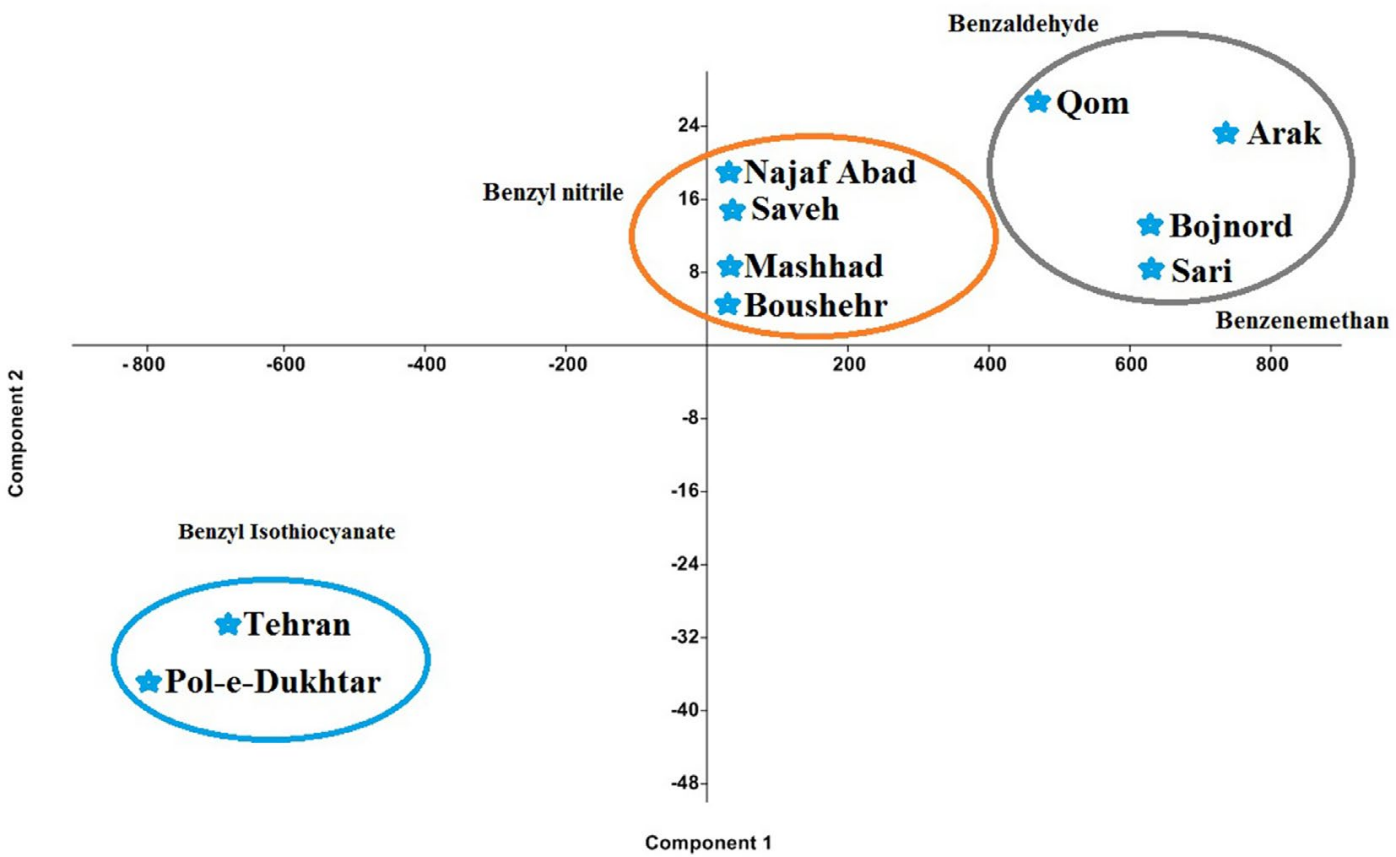
The PCA plot of essential oils data demonstrated the existence of three chemotypes among the evaluated populations.

Principal component analysis (PCA) generated five principal components that together explained more than 99% of the total variance in the quantitative dataset. The first two principal components were used to construct the PCA plot (Figure 3), revealing three chemotype groups. The first principal component (PC1) accounted for 56% of the total variance and was mainly associated with variations in benzyl nitrile and benzyl isothiocyanate concentrations. The second principal component (PC2) explained 33% of the total variance and was primarily related to benzaldehyde and benzenemethanethiol concentrations.

Accordingly, the Arak and Pol‐e‐Dokhtar populations were identified as the Benzyl isothiocyanate chemotype group, whereas the Najaf Abad, Saveh, Mashhad, and Bushehr populations formed the benzyl nitrile chemotype group. The remaining populations were characterized by relatively higher amounts of benzaldehyde and benzenemethanethiol.

**FIGURE 4:**
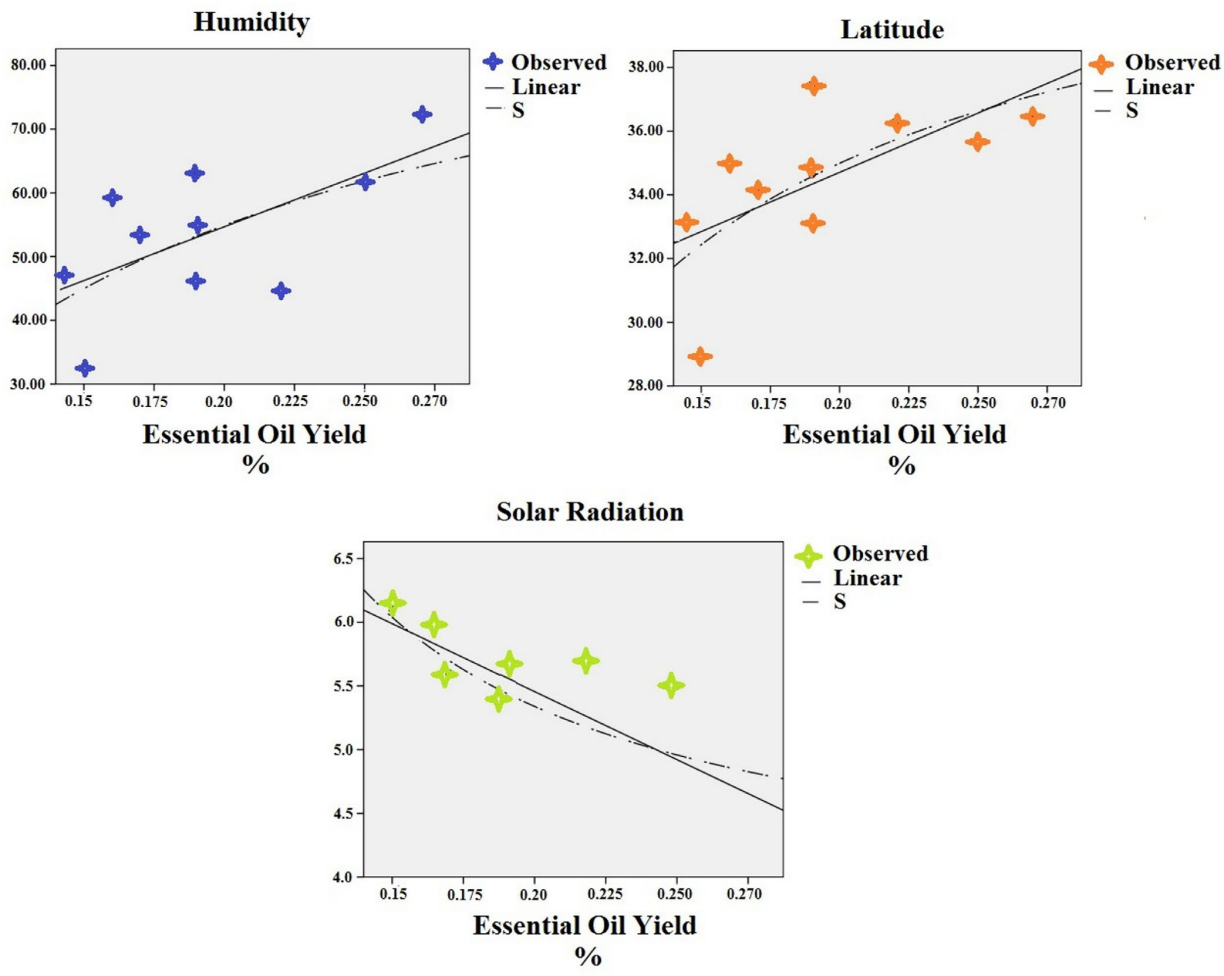
Diagrams exhibit significant correlations between essential oil yield and some environmental parameters.

Overall, the essential oil composition of the studied populations was characterized by low yield and hydrocarbon dominance, with minor exceptions. Benzyl nitrile was consistently the principal constituent across all populations, while the secondary major constituents varied among populations. No significant differences (*p* < 0.05) were observed in the major essential oil constituents among the studied populations, suggesting a relatively stable chemical composition across different habitats. However, essential oil yield was significantly influenced by environmental factors. A positive correlation was found between essential oil yield and habitat humidity (*p* ≤ 0.05, *r* = 0.63), indicating that higher moisture availability may promote essential oil production. In contrast, solar radiation showed a significant negative correlation with oil yield (*p* ≤ 0.01, *r* = −0.78), suggesting that increased radiation intensity may reduce essential oil accumulation. Additionally, populations distributed in northern regions exhibited significantly higher essential oil yields (*p* ≤ 0.05, *r* = 0.64), likely due to more favorable ecological conditions (Figure 4). Despite these environmental influences on oil yield, no significant relationships were detected between the major essential oil constituents and geographic location, climatic variables, or phytogeographic distribution. The only exception involved the Najaf Abad and Saveh populations, which belonged to the same phytogeographic district and displayed close clustering.

### 3.3 Seed Oil Fatty Acid Profile

The seed oil fatty acid profiles of the evaluated *L. sativum* populations are presented in Table 4. The oils consisted of both saturated and unsaturated fatty acids, with unsaturated‐to‐saturated fatty acid ratios ranging from 4.47 in the Saveh population to 5.42 in the Mashhad population. Among the studied populations, the highest proportion of total saturated fatty acids was recorded in the Saveh population (18.25%), whereas the lowest was observed in the Mashhad population (15.5%).

**TABLE 4:** Fatty acid composition of seed oil in the evaluated populations of *L. sativum* . All fatty acid contents are expressed as percentages of total fatty acids.

Fatty acid	Formula	Bushehr	Saveh	Najaf Abad	Mashhad	Tehran	Pol‐e‐Dokhtar	Qom	Arak	Sari	Bojnord
Myristic	C14:0	0.11	0.14	0.11	0.12	0.08	0.07	0.14	0.11	0.12	0.08
Palmitic	C16:0	9.28	10.57	9.87	9.75	8.35	9.37	9.28	9.02	9.07	8.12
Margaric	C17:0	0.04	0.05	0.04	0.03	0.04	0.05	0.04	0.04	0.04	0.04
Stearic	C18:0	3.47	3.44	3.37	3.25	3.15	3.49	3.08	3.20	3.55	3.25
Arachidic	C20:0	3.21	2.92	2.21	1.19	2.99	2.99	2.92	3.02	3.13	3.07
Behenic	C22:0	0.80	0.73	0.91	0.75	0.73	0.74	0.80	0.76	0.84	0.76
Lignoceric	C24:0	0.41	0.40	0.47	0.47	0.44	0.47	0.54	0.51	0.51	0.47
Total Saturated fatty acids		17.32	18.25	16.98	15.56	15.78	17.18	16.80	16.66	17.26	15.79
Myristoleic	C14:1 (5c)	0.02	0.03	0.02	0.03	0.02	0.03	0.03	0.03	0.03	0.02
Palmitoleic	C16:1 (7c)	0.24	0.28	0.25	0.25	0.24	0.27	0.29	0.25	0.26	0.23
Heptadecenoic	C17:1 (10c)	0.06	0.04	0.03	0.03	0.03	0.04	0.08	0.05	0.06	0.03
oleic	C18:1 (9c)	25.79	25.42	25.76	27.96	26.00	29.08	26.65	23.67	26.30	23.86
Linoleic	C18:2 (9c, 12c)	11.74	12.46	11.92	13.10	12.22	12.49	11.62	12.55	12.59	12.66
α‐Linolenic	C18:3 (9c, 12c, 15c)	28.55	27.70	27.61	28.32	30.61	27.28	30.02	31.69	28.69	32.38
Eicosenoic	C20:1 (9c)	11.28	10.65	11.03	10.14	10.77	9.91	10.07	10.52	10.52	10.63
Eicosadienoic	C20:2 (11c, 14c)	0.43	0.42	0.50	0.44	0.45	0.37	0.31	0.46	0.41	0.47
Erucic	C22:1 (9c)	3.81	3.94	5.04	3.33	3.15	2.74	3.27	3.27	3.05	3.13
	C24:1	0.71	0.76	0.84	0.80	0.71	0.58	0.83	0.82	0.80	0.75
MUFAs/PUFAs ratio		1.02	1.01	1.07	1.01	0.94	1.06	0.98	0.86	0.98	0.84
Total unsaturated fatty acids		82.63	81.70	83.00	84.40	84.20	82.79	83.17	83.31	82.71	84.16
UFA/SFA ratio		4.77	4.47	4.88	5.42	5.33	4.81	4.95	5.00	4.79	5.32
Antioxidant activity (ppm)**		1109.13 ± 9.13	857.48 ± 4.25	932.91 ± 1.6	831.93 ± 7.09	876.97 ± 4.19	749.75 ± 4.09	918.49 ± 2.55	843.32 ± 5.03	804.74 ± 3.92	842.60 ± 8.42

*Note:* Asterisks (**) show significant variation by ANOVA test. Abbreviations: (c), *cis* configuration of double bond(s); C, carbon number of each fatty acid; MUFAs, monounsaturated fatty acids; ppm, parts per million; PUFAs, polyunsaturated fatty acids; SFAs, saturated fatty acids; UFAs, unsaturated fatty acids.

Palmitic acid was identified as the predominant saturated fatty acid, reaching its highest level in the Saveh population (10.57%) and its lowest in the Bojnurd population (8.12%). Stearic acid, the second most abundant saturated fatty acid, varied from 3.08% in the Qom population to 3.55% in the Sari population. In addition, trace amounts of other saturated fatty acids were detected across all populations.

The total unsaturated fatty acid content was 81.70% in the Saveh population, whereas a slightly higher value of 84.40% was recorded in the Mashhad population. α‐Linolenic acid, identified as the principal unsaturated fatty acid, reached its highest proportion in the Bojnord population (32.38%) and its lowest in the Najaf Abad population (27.61%). Oleic acid, the second most abundant unsaturated fatty acid, reached its highest level in the Pol‐e‐Dukhtar population (29.08%) and its lowest level in the Arak population (23.67%).

Linoleic acid (11.62%–13.10%) and eicosenoic acid (9.91%–11.28%) were identified as the third and fourth most abundant unsaturated fatty acids, respectively. In addition, several other unsaturated fatty acids were detected in trace amounts across the examined populations.

The unsaturated‐to‐saturated fatty acid ratio ranged from 4.47 in the Saveh population to 5.42 in the Mashhad population, indicating that unsaturated fatty acids were approximately five times more abundant than saturated fatty acids in the evaluated populations.

Overall, α‐linolenic acid exhibited approximately 5% variation among the studied populations, ranging from 27.61% to 32.38%. However, no significant differences were observed among populations with respect to α‐linolenic and oleic acid contents.

**FIGURE 5:**
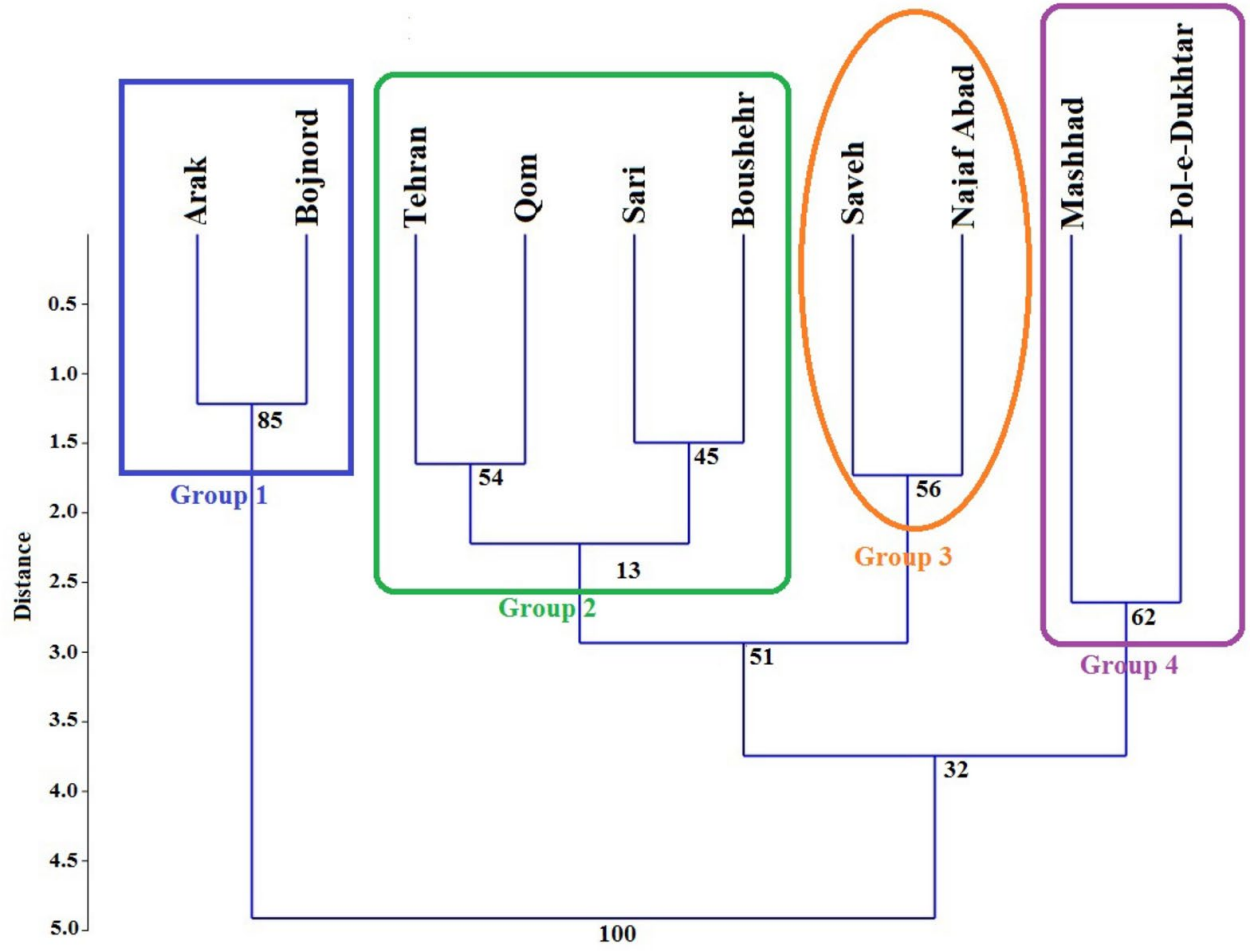
The UPGMA dendrogram of the examined *L. sativum* populations constructed on seed oil fatty acids profile (number below each node exhibits a bootstrapping value).

**FIGURE 6:**
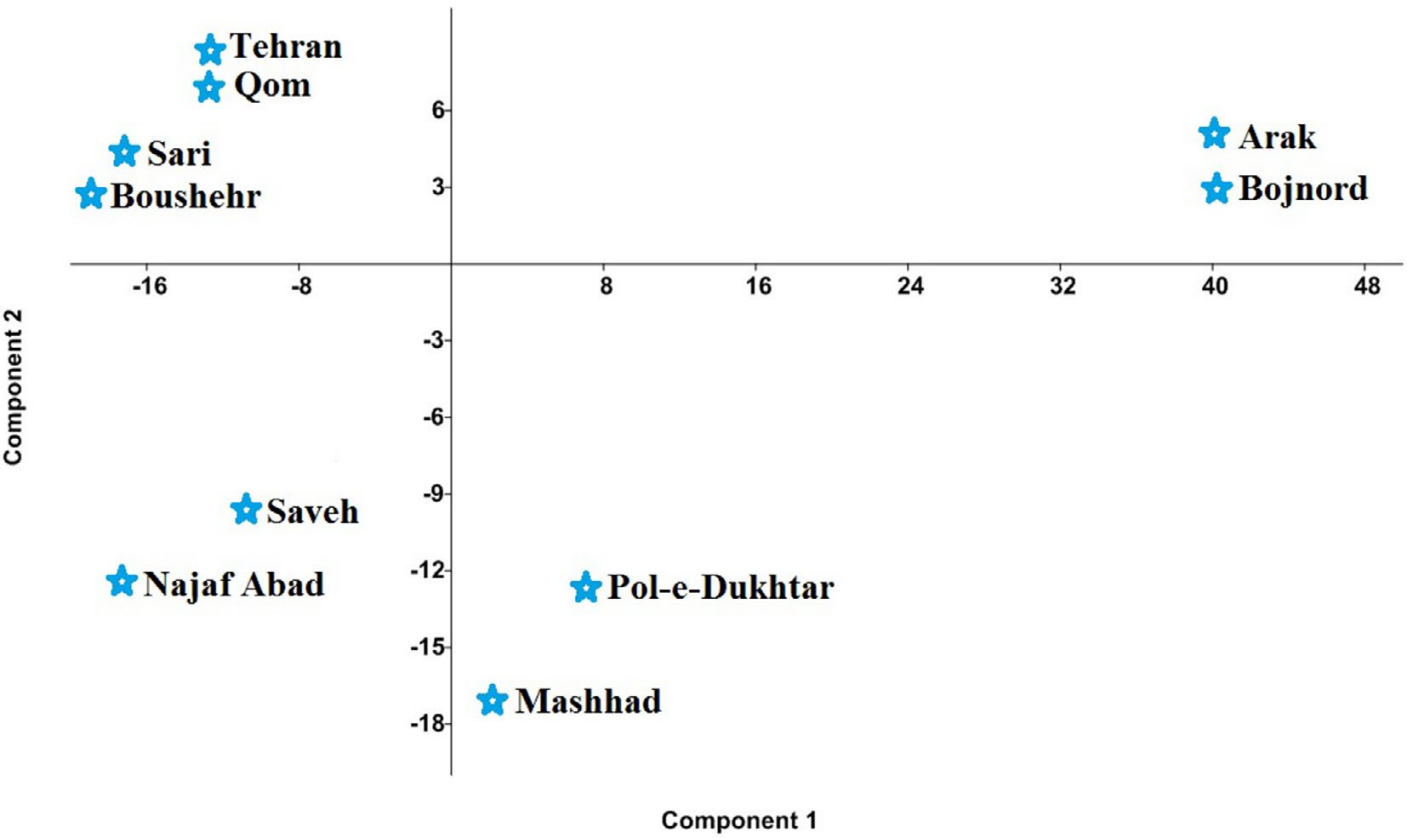
The PCA plot of the investigated *L. sativum* populations, based on the seed oil fatty acid composition.

Based on the UPGMA dendrogram (Figure 5) and PCA ordination (Figure 6), the evaluated populations were grouped into four distinct clusters: (1) the Arak and Bojnord populations formed a cluster with 100% bootstrap support, characterized by high α‐linolenic acid and low oleic acid contents; (2) the Tehran, Qom, Sari, and Bushehr populations were closely associated; (3) the Saveh and Najaf Abad populations constituted a separate group characterized by high erucic acid content; and (4) the Mashhad and Pol‐e‐Dukhtar populations formed another distinct cluster with elevated oleic acid levels.

No significant association was observed between the major fatty acid composition and geographic origin, climatic conditions, or phytogeographic distribution of the populations. An exception was noted for the Najaf Abad and Saveh populations, which belonged to the same phytogeographic district and clustered closely, suggesting a degree of phytogeographic affinity.

### 3.4 DPPH Radical Scavenging Activity for Seed Oils

The IC_50_ value for DPPH radical scavenging activity—defined as the concentration required to achieve 50% inhibition—averaged 876.32 ppm across populations. Observed values ranged from 749.75 ± 4.09 ppm in the Pol‐e‐Dokhtar population to 1109.32 ± 9.13 ppm in the Bushehr population (Table 4). Although one‐way ANOVA indicated a statistically significant effect of population on antioxidant activity (*p* < 0.001), subsequent post hoc analyses (Duncan's multiple range test, Tukey B, and Tukey's HSD) did not reveal significant pairwise differences among populations (Table 3), suggesting that the overall variance detected by ANOVA was not attributable to distinct group separations.

## 4 Discussion

### 4.1 Essential Oil Yield, Composition, and Biological Relevance

The essential oil yield of the examined populations ranged from 0.14% to 0.27% (v/w), reflecting the generally low essential oil productivity of *L. sativum* . This result is consistent with the findings of Mirza and Najafpour‐Navaei (2006), who reported an essential oil yield of 0.3% (v/w) for an Iranian population of the species. Nevertheless, variation in essential oil yield among the evaluated populations appeared to be associated with ecological conditions, particularly humidity, solar radiation, and northern geographical distribution, suggesting that environmental factors play an important role in modulating essential oil biosynthesis in *L. sativum* . Previous studies have shown that environmental parameters, including water availability (Costa et al. 2014; Castello et al. 2012), latitude, and solar radiation, can markedly influence essential oil yield in plants; however, the direction and magnitude of these effects are species‐dependent.

Essential oil analysis of seeds revealed a high level of chemical diversity, with benzyl nitrile and benzyl isothiocyanate identified as the two major compounds. This finding also highlighted a wide range variation in benzyl nitrile levels (over 45%) between different populations. According to Lee and Lee (2025), benzyl nitrile was the main component of essential oil in some *Lepidium* species, including *Lepidium meyenii* where it constitutes 71.64% of the essential oil compounds. This compound exhibits potent insecticidal activity against several insect vectors responsible for transmitting various pathogenic viruses. Benzyl nitrile is a toxic volatile compound found in some plants, like *Camellia sinensis* (L.) Kuntze, that accumulates under stress and acts as a defense mechanism (Liao et al. 2020). It is derived from L‐phenylalanine via phenylacetaldoxime and some enzymes such as CsCYP79D73 and CsCYP71AT96s are involved in this process. Biosynthesis of benzyl nitrile is linked to the activation of defense‐related phytohormones like jasmonic acid (Liao et al. 2020). Additionally, Qian et al. (2023) suggested that abiotic (e.g., light exposure) and biotic (e.g., herbivore attacks) parameters can trigger the biosynthesis of benzyl nitrile, likely as a defense mechanism.

Benzyl isothiocyanate, the second significant component in the essential oils of some populations, along with other isothiocyanate derivatives (such as allyl isothiocyanate, benzyl isothiocyanate, phenethyl isothiocyanate, and sulforaphane) is naturally present in various species belonging to the Brassicaceae family. Isothiocyanates (ITCs) possess a highly reactive electrophilic carbon atom within their N = C = S structures, which upon hydrolysis can generate reactive oxygen species (ROS) and lead to oxidative DNA damage. These compounds have demonstrated various biological properties, including anti‐inflammatory, antibacterial, anticancer, and antifungal effects (Kasiappan et al. 2016; Dinh et al. 2021).

Additionally, the roots of *Salvadora persica* L. (Salvadoraceae) contain significant amounts of benzyl isothiocyanate (39.4%) and benzyl nitrile (37.9%). These compounds have demonstrated potent antimicrobial activity against various plant pathogenic microorganisms such as *Agrobacterium tumefaciens* , *Pectobacterium atrosepticum* , *Enterobacter cloacae* , *Dickeya solani*, *Ralstonia solanacearum* , and some human bacteria, including: *Bacillus subtilis* , *Sarcina lutea*, *Escherichia coli* , and *Staphylococcus aureus* (Scotti and Barlow 2022; Salem et al. 2018). The antibacterial properties of garden cress essential oil, particularly the seed extract, suggest its potential use in treating bacterial infections in both humans and plants.

Benzaldehyde was one of the oil components and it can reach percentages as high as 6% or even higher. It is widely used in the food, beverage, and fragrance industries due to its almond‐like aroma and flavor. This compound also has various biological properties (such as anti‐tumor, antibacterial, and antifungal) and is found in high concentrations (63.1%–98.3%) in the essential oils of some plants, particularly *Prunus persica* (L.) Batsch (Verma et al. 2017).

Heneicosane was detected in both the Qom and Tehran populations, but at different concentrations, indicating the presence of a shared chemical constituent between these populations. Vanitha et al. (2020) examined the leaf extract biological activities of *Plumbago zeylanica* L., and reported excellent antibacterial activities of heneicosane, as the main compound, against *Streptococcus pneumoniae* and *Aspergillus fumigatus* . Therefore, the essential oil of these populations, particularly the Qom population, may have potential for inhibiting the growth of certain pathogenic bacteria.

Jelvehgar et al. (2024) analyzed the essential oil composition of *L. sativum* seeds from seven Iranian regions, finding 1,8‐cineole (44.5%), α‐terpinyl acetate (13.9%), and α‐pinene (7.7%) as major components. The study also noted variations in these components across different populations. However, no similarity was found between their findings and the results of the current study.

Getahun et al. (2020) examined the seed essential oils of *L. sativum* plant, which were extracted using diverse extraction methods. They detected 1‐isocyano‐2‐methylbenzene (71.63%) and benzaldehyde (11.21%) in the oil extracted using a Clevenger‐type apparatus. Meanwhile, the oil extracted by a modified simple distillation method had 1‐isocyano‐2‐methylbenzene (36.18%), benzyl isothiocyanate (8.71%), and benzaldehyde (2.96%) as the main compounds.

In this study, the essential oils extracted using a Cleavinger‐type apparatus have a chemical composition very similar to those obtained using a modified simple distillation method, as described by Getahun et al. (2020). This suggests that both methods yield comparable results in terms of the types and proportions of volatile compounds extracted from the seeds material.

The composition of essential oils is influenced by various factors, including harvesting time, extraction methods, genetic structure, environmental conditions, and geographic location. These factors can significantly affect the types and amounts of chemical compounds present in the essential oils (Talebi et al. 2020; Maurya et al. 2018). In this sense, geographic coordinates (such as altitude, latitude, and longitude), biotic (herbivory and pathogens), and even abiotic (climate and soil type) stresses can influence the plant's metabolism and consequently the essential oil it produces (Qaderi et al. 2023). The analysis of essential oils from different Iranian garden cress populations revealed no significant correlation between the main oil constituents and the geographic locations, climatic features, and phytogeographic distribution of their habitats. This finding is consistent with a previous study on essential oils from various Iranian *L. sativum* populations (Jelvehgar et al. 2024).

### 4.2 Fatty Acid Profile and Environmental Influence

This study revealed a 3% variation in saturated fatty acid content among populations of *L. sativum* , suggesting that environmental factors have limited influence on saturated fatty acid composition. This finding agreed with a previous study by Chatoui et al. (2020), which examined the seed oil fatty acids profile of four *L. sativum* populations in Morocco and detected nearly equal percentages of saturated fatty acids among the populations.

The study indicated a slight variation (approximately 2.5%) in total unsaturated fatty acids percentage across the populations. This result is consistent with a previous study on *L. sativum* seed oils by (Chatoui et al. 2020). Additionally, Gokavi et al. (2004) reported that different extraction methods for garden cress seed oil yielded similar total amounts of unsaturated fatty acids, suggesting that the extraction method does not significantly influence the overall fatty acid profile. This finding is consistent with the present study, where the total saturated and unsaturated fatty acid contents in some populations (Mashhad, Tehran, and Bojnord) were comparable to those reported by Gokavi et al. (2004).

α‐Linolenic acid, the primary fatty acid, showed approximately 5% variation among the populations. This finding aligns with previous studies like those by Gokavi et al. (2004) and Chatoui et al. (2020), which also identified α‐linolenic acid as the primary component in *L. sativum* seed oil, though the exact percentages varied across the mentioned studies.

Chatoui et al. (2020) found that variations in fatty acid content were not linked to different extraction methods. However, they observed a small difference in the amount of a specific fatty acid among Moroccan populations, contrary to the findings of this study. While, according to Angelini et al. (1997), oleic acid (29.4%–35.6%) was identified as the primary fatty acid in *L. sativum* seed oil, with linolenic acid (20.4%–29.5%) being the second most abundant.

α‐linolenic acid, an essential omega‐3 fatty acid, is a precursor to longer‐chain omega‐3 fatty acids like eicosapentaenoic acid and docosahexaenoic acid. While the conversion of α‐linolenic acid to eicosapentaenoic and docosahexaenoic acids is limited, these fatty acids possess important biological properties, including anti‐inflammatory and pro‐resolution effects (Anderson and Ma 2009; Cambiaggi et al. 2023).

Additionally, it seems that α‐linolenic acid supplementation in the human diet improves lipid profile, reduces the inflammatory biomarker C‐reactive protein, decreases cardiovascular diseases, and also all‐cause mortality (Cambiaggi et al. 2023).

Oleic acid was identified as the second most abundant fatty acid, showing approximately 6% variation among populations. Previous studies by Chatoui et al. (2020) and Gokavi et al. (2004) reported similar levels (23%–24.08%) in Moroccan (Chatoui et al. 2020) and Bangladesh populations (Gokavi et al. 2004). In this study, some populations showed comparable values, while most exhibited higher oleic acid content than previously reported.

Contrary to the present findings, Chatoui et al. (2020) reported no prominent infraspecific variation in oleic acid among Moroccan populations. In contrast, only trace amounts of oleic acid were detected in the Saudi Arabian population of *L. sativum* (Alqahtani et al. 2018). Additionally, several major fatty acids identified in the Saudi population seed oil profile—including 7,10,13‐hexadecatrienoic acid (44.37%), 11‐octadecenoic acid (15.50%), behenic acid (9.67%), and 15‐tetracosenoic acid (5.68%)—were not detected in the Iranian, Bangladeshi, and Moroccan populations.

The climatic, phytogeographical, and geographic data indicate substantial environmental variation across sampling locations. In particular, there is a 1700 m difference in altitude between the lowest and highest habitats, along with marked differences in mean annual temperature and precipitation. The populations also originate from two distinct phytogeographical regions and, even within the same region, are distributed across diverse districts, indicating a heterogeneous spatial pattern. Overall, these populations occupy ecologically diverse niches under varying climatic conditions, with greater environmental heterogeneity than that reported for Moroccan populations Chatoui et al. (2020).

Sabbaghnia et al. (2024) reported morphological divergence among diverse populations of *L. sativum* , which may account for the limited chemical variability observed in the present study. Although environmental factors significantly influence the seed oil fatty acid profile of this species, genetic structure and breeding practices also play an important role in determining oil composition Talebi et al. (2025).

Antioxidant activity was evaluated based on IC50 values, defined as the concentration required to inhibit 50% of a biological process. Activity was classified as very strong (IC50 < 50 μg/mL), strong (50–100 μg/mL), moderate (101–250 μg/mL), weak (250–500 μg/mL), and inactive (≥ 500 μg/mL), with lower IC50 values indicating higher antioxidant potency (Jumina et al. 2019). In this study, the minimum IC50 value of the seed oil was approximately 750 ppm, indicating that all examined populations exhibited very weak to negligible antioxidant activity.

Although one‐way ANOVA indicated a statistically significant difference in antioxidant activity among populations, post hoc multiple comparison tests did not reveal significant pairwise differences. This discrepancy may arise when the overall F‐test detects collective variation among group means relative to within‐group variability, while no individual pairwise comparison remains significant after correction for multiple testing. Such outcomes typically reflect small, distributed differences across groups rather than clear separation between specific population pairs (Kim 2015).

Previous studies by Ballus et al. (2015) and Kanabur (2022) reported that garden cress seed extracts and seed oil exhibit strong antioxidant activity, with high total polyphenol content and notable ORAC (Oxygen Radical Absorbance Capacity) values, as well as potent DPPH radical scavenging activity. These results suggest their potential as natural antioxidant sources for various applications. However, the findings of the present study do not fully align with these earlier reports.

### 4.3 Chemotype Grouping and Population Variability

Clustering analysis revealed three chemotype groups based on essential oil composition in the evaluated populations. One group is characterized by a high concentration (over 95%) of benzyl nitrile. Another group has benzyl nitrile as the main component (around 50%), with benzyl isothiocyanate also present at over 40%. The third group features benzyl nitrile as the dominant compound (68%–81%), along with other bioactive compounds in significant percentages. Previous molecular genetic diversity studies using RAPD (Random Amplified Polymorphic DNA‐ Bansal et al. 2012) and ISSR (Inter‐Simple Sequence Repeat‐ Kaur et al. 2015; Kumar and Yadav 2019) markers have revealed significant genetic divergence and the presence of distinct genotypes among various populations of *L. sativum* . While Saveh and Najaf Abad populations grouped close together based on shared origins within the same phytogeographic district, other populations did not exhibit a clear clustering pattern based on geographic location, climate conditions, or phytogeographic distribution.

This study exhibited that the investigated *L. sativum* populations could be grouped into four distinct clusters based on variations in their seed oil fatty acids composition but did not identify any distinct chemotypes. This suggests a relatively uniform fatty acids profile across the populations despite the observed clustering pattern. While most populations did not show a clear clustering pattern based on geographic location, climate conditions, or phytogeographic distribution, Saveh and Najaf Abad populations clustered together based on their shared origin within the same phytogeographic district. Roughani et al. (2018) examined agro‐morphological traits of six Iranian garden cress populations and classified them in three major clusters.

Comparison of population clustering patterns in the seed oil and essential oil UPGMA dendrograms revealed a single consistent pattern, with the Saveh and Najaf Abad populations clustering closely in both analyses.

From an ecological perspective, the variation observed among *L. sativum* populations appears to reflect the influence of environmental conditions on metabolite production rather than on chemical composition. Essential oil yield was positively associated with some ecological parameters. In contrast, the major essential oil constituents showed relatively low variation among populations and were not significantly correlated with climatic or geographic variables, indicating that the biosynthesis of these compounds is likely under stronger genetic control. Such chemical stability may represent an adaptive ecological strategy because these compounds play important roles in plant defense against herbivores, pathogens, and environmental stress. Furthermore, the close clustering of the Saveh and Najaf Abad populations likely reflects similar ecological and phytogeographic conditions that may promote comparable metabolic responses and chemical profiles.

This study has some limitations. The essential oil yield was very low, which may have affected the detection of minor compounds. Sampling was conducted at a single time point, without considering seasonal or developmental variations that may influence metabolite composition. In addition, the lack of molecular data limits the interpretation of chemotypic differences among populations. The antioxidant activity was evaluated only using the DPPH assay, which provides a limited assessment of antioxidant potential. Future studies should include multi‐seasonal sampling, larger population sizes, and molecular approaches to better understand the genetic basis of chemical variation. Additional antioxidant assays, such as β‐carotene bleaching and FRAP, are recommended to provide a more comprehensive evaluation of antioxidant capacity. Controlled experiments are also needed to distinguish environmental and genetic effects on metabolite composition. Future investigations should incorporate a battery of complementary antioxidant assays—including FRAP, ABTS, and β‐carotene bleaching tests—to elucidate the diverse mechanistic pathways underlying the antioxidant activity of *L. sativum* seed oils. Additionally, correlating antioxidant capacity with specific phytochemical constituents (e.g., phenolics, tocopherols, glucosinolates) through HPLC or LC–MS profiling would enhance the interpretability of structure–activity relationships.

## 5 Conclusion

This study provides a comprehensive evaluation of essential oil yield, chemical composition, fatty acid profile, and antioxidant activity in *L. sativum* populations across different ecological regions. The results demonstrated generally low essential oil productivity that is consistent with previous reports for this species. Despite this low yield, variation among populations was observed, indicating that environmental conditions play an important role in regulating essential oil biosynthesis. In particular, significant positive/negative correlations were detected between oil yield and some ecological parameters, highlighting the sensitivity of secondary metabolite production to environmental parameters. Chemical profiling revealed considerable diversity in seed essential oil composition, with benzyl nitrile and benzyl isothiocyanate identified as the dominant constituents. The variability in benzyl nitrile content among populations suggests adaptive or stress‐related metabolic regulation, potentially linked to plant defense mechanisms. These compounds are known for their significant biological activities, reinforcing the ecological and pharmacological relevance of *L. sativum* essential oils. However, despite this chemical variability, no consistent relationship was observed between major constituents and geographic, climatic, or phytogeographic factors, suggesting a relatively stable chemotypic framework across populations. Fatty acid analysis indicated only slight variation among populations, with α‐linolenic acid as the predominant fatty acid, followed by oleic acid. This stability suggests that fatty acid biosynthesis in *L. sativum* seeds is less influenced by environmental variation compared to volatile secondary metabolites. Nonetheless, the nutritional significance of α‐linolenic acid highlights the potential health value of the species. Antioxidant assays revealed weak overall activity across all populations, with no clear separation among groups despite statistical differences. This contrasts with previous reports of strong antioxidant potential, likely due to methodological differences or environmental influences on phytochemical accumulation. Cluster analysis identified distinct chemotype groupings based on essential oil composition, although fatty acid profiles remained relatively uniform. These findings may guide future breeding programs, promote the use of *L. sativum* essential oils in natural pest control, and support the development of eco‐friendly industrial products.

## Author Contributions

**Ghassan Mohammad:** investigation, software, data curation. **Majid Mahdieh:** conceptualization, supervision, resources, writing – original draft, methodology. **Mansour Ghorbanpour:** writing – review and editing, software, validation, methodology. **Seyed Mehdi Talebi:** conceptualization, formal analysis, writing – original draft, software, data curation, methodology.

## Funding

The authors have nothing to report.

## Ethics Statement

The authors have nothing to report.

## Consent

We hereby declare that we participated in this study and the manuscript's development. The final version of the paper has been checked and given consent for publication.

## Conflicts of Interest

The authors declare no conflicts of interest.

## Data Availability

The data that support the findings of this study are available on request from the corresponding author. The data are not publicly available due to privacy or ethical restrictions.
